# Supplementary material for: Increases in reef size, habitat and metacommunity complexity associated with Cambrian radiation oxygenation pulses
Source: Nat Commun. 2022 Dec 6;13:7523. doi: 10.1038/s41467-022-35283-5 (PMC9727068; doi:10.1038/s41467-022-35283-5)
Supplement: Supplementary file 1 — Supplementary Information [file 41467_2022_35283_MOESM1_ESM.pdf]

Supplementary Information for

Increases in reef size, habitat, and metacommunity complexity associated with Cambrian Radiation oxygenation pulses

Andrey Yu. Zhuravlev, Emily G. Mitchell, Fred Bowyer, Rachel Wood and Amelia Penny

Correspondence to:

Andrey Zhuravlev
ayzhur@mail.ru

Emily Mitchell
ek338@cam.ac.uk

Supplementary Table 1 | Reef occupation based on Zones 1-4 (relative water depth) on the Aldan and middle Lena rivers, Siberian Platform, based on Siberian stratigraphic stages and correlation with carbon isotope excursions (5p – VII).

Age	Zone 1	Zone 2	Zone 3	Zone 4
Nemakit-Daldynian 4 /below 6p/7p		X		
Tommotian 1 / below II		X		
Tommotian 2 / II		X		
Tommotian 3 / III		X		
Tommotian 4 / below IV		X		
Atdabanian 1 / IV	X	X	X	
Atdabanian 2 / below V	?	X	X	
Atdabanian 3 / below VI	?	X	X	
Atdabanian 4 /VI	X	X	X	X
Botoman 1 / VII	X	X	X	X

Supplementary Table 2 | Reef/bioherm size (diameter) in Zones 1-4 (relative water depth) on the Aldan and middle Lena rivers, Siberian Platform, based on Siberian stratigraphic stages and correlation with carbon isotope excursions (5p – VII).

Age	Zone 1	Zone 2	Zone 3	Zone 4
Nemakit-Daldynian 4 /below 6p/7p		No reefs		
Tommotian 1 / below II		1.7 /ref.3		
Tommotian 2 / II		0.4-3.0/ ref.1,6-8		
Tommotian 3 /III		0.5-2.0/ ref.1,6,8		
Tommotian 4 / below IV		0.5-1.0/ ref.1,6,8		
Atdabanian 1 / IV	No reefs	1.0-6.0/ ref.2,4,6-8	0.5-1.5/ ref.5	
Atdabanian 2 / below V	?	1.0-6.0/ ref.2,4,6-8	0.1-1.0/ ref.4,5,6,9	
Atdabanian 3 / below VI	?	No reefs	0.1-0.5/ ref.5	
Atdabanian 4 / VI	No reefs	No data	0.2-1.0/ ref.5,6,8	0.3/ ref.5
Botoman 1 / VII	No reefs	No data	0.2-1.0/ ref.5,6,8	0.3/ ref.5

Supplementary Table 3: Number of reef community types, based on Siberian stratigraphic stages and correlation with carbon isotope excursions (5p – VII), calculated from Supplementary Data 1.

**Age**	**Community type numbers**
Nemakit-Daldynian 4 /below 6p/7p	1
Tommotian 1 / below II	2
Tommotian 2 / II	12
Tommotian 3 / III	6
Tommotian 4 / below IV	6
Atdabanian 1 / IV	12
Atdabanian 2 / below V	5
Atdabanian 3 / below VI	5
Atdabanian 4 / VI	8
Botoman 1 / VII	8

Supplementary References

1. Kruse, P. D., Zhuravlev, A. Yu. & James, N. P. Primordial metazoan-calcimicrobial reefs: Tommotian (Early Cambrian) of the Siberian Platform. *Palaios* **10**, 291–321 (1995).

2. Kruse, P. D., Zhuravlev, A. Yu. & James, N. P. Field data of 1989.

3. Riding, R. & Zhuravlev, A. Yu. Structure and diversity of oldest sponge-microbe reefs: Lower Cambrian, Aldan River, Siberia. *Geology* **23**, 649–652 (1995).

4. Rowland, S. M. & Gangloff, R. A. Structure and paleoecology of Lower Cambrian reefs. *Palaios* **3**, 111–135 (1988).

5. Zhuravlev, A. Yu. Field data of 1978–1987.

6. Zhuravleva, I. T. in *Organism and Environment in the Geological Past* (ed. Gekker, R. F.) 61–84 (Nauka, 1966) [in Russian].

7. Zhuravleva, I. T. in *Problems of Lower Cambrian Biostratigraphy and Palaeontology of Siberia* (ed. Zhuravleva, I. T.) 31–109 (Nauka, 1972) [in Russian].

8. Zhuravleva, I. T. & Zelenov, K. K. Bioherms of the Pestrotsvet Formation of the Lena River. *Trans. Paleontol. Inst. USSR Acad. Sci*. **56**, 57–77 (1955) [in Russian].

9. Zhuravlev, A. Yu. & Naimark, E. B. Alpha, beta, or gamma: Numerical view on the Early Cambrian world. *Palaeogeogr. Palaeoclimatol. Palaeoecol*. 220, 207–225 (2005).
