# Supplementary material for: Increases in reef size, habitat and metacommunity complexity associated with Cambrian radiation oxygenation pulses
Source: Nat Commun. 2022 Dec 6;13:7523. doi: 10.1038/s41467-022-35283-5 (PMC9727068; doi:10.1038/s41467-022-35283-5)
Supplement: Supplementary file 3 — Description of Additional Supplementary Files [file 41467_2022_35283_MOESM3_ESM.pdf]

Description of Additional Supplementary Materials

Supplementary Data 1: Early Cambrian (Tommotian/Stage 2 – Atdabanian/Stage 3) reef palaeocommunities of the Siberian Platform listed from the shallowest to the deepest (stratigraphic distribution is given in brackets).

Supplementary Code 1: Code to complete metacommunity analyses, as well as code for calculating diversity, coverage-standardised species richness through time, coverage-standardised species richness, and observed richness.
