# Supplementary material for: Increases in reef size, habitat and metacommunity complexity associated with Cambrian radiation oxygenation pulses
Source: Nat Commun. 2022 Dec 6;13:7523. doi: 10.1038/s41467-022-35283-5 (PMC9727068; doi:10.1038/s41467-022-35283-5)
Supplement: Supplementary file 6 — Reporting Summary [file 41467_2022_35283_MOESM6_ESM.pdf]

nature portfolio

Corresponding author(s): Emily Mitchell

Last updated by author(s): Nov 21, 2022

Reporting Summary

Nature Portfolio wishes to improve the reproducibility of the work that we publish. This form provides structure for consistency and transparency in reporting. For further information on Nature Portfolio policies, see our Editorial Policies and the Editorial Policy Checklist.

Statistics

For all statistical analyses, confirm that the following items are present in the figure legend, table legend, main text, or Methods section.

n/a	Confirmed	
☐	☒	The exact sample size (*n*) for each experimental group/condition, given as a discrete number and unit of measurement
☒	☐	A statement on whether measurements were taken from distinct samples or whether the same sample was measured repeatedly
☒	☐	The statistical test(s) used AND whether they are one- or two-sided<br>*Only common tests should be described solely by name; describe more complex techniques in the Methods section.*
☒	☐	A description of all covariates tested
☒	☐	A description of any assumptions or corrections, such as tests of normality and adjustment for multiple comparisons
☐	☒	A full description of the statistical parameters including central tendency (e.g. means) or other basic estimates (e.g. regression coefficient) AND variation (e.g. standard deviation) or associated estimates of uncertainty (e.g. confidence intervals)
☒	☐	For null hypothesis testing, the test statistic (e.g. *F*, *t*, *r*) with confidence intervals, effect sizes, degrees of freedom and *P* value noted<br>*Give P values as exact values whenever suitable.*
☒	☐	For Bayesian analysis, information on the choice of priors and Markov chain Monte Carlo settings
☒	☐	For hierarchical and complex designs, identification of the appropriate level for tests and full reporting of outcomes
☒	☐	Estimates of effect sizes (e.g. Cohen's *d*, Pearson's *r*), indicating how they were calculated

Our web collection on statistics for biologists contains articles on many of the points above.

Software and code

Policy information about availability of computer code

Data collection	*Provide a description of all commercial, open source and custom code used to collect the data in this study, specifying the version used OR state that no software was used.*
Data analysis	Analyses were performed in R v4.1.1 using the packages metacom v2.5.7, cooccur v1.2 and iNext v3.0.0

For manuscripts utilizing custom algorithms or software that are central to the research but not yet described in published literature, software must be made available to editors and reviewers. We strongly encourage code deposition in a community repository (e.g. GitHub). See the Nature Portfolio guidelines for submitting code & software for further information.

Data

Policy information about availability of data

All manuscripts must include a data availability statement. This statement should provide the following information, where applicable:

- Accession codes, unique identifiers, or web links for publicly available datasets
- A description of any restrictions on data availability
- For clinical datasets or third party data, please ensure that the statement adheres to our policy

Code and data are included as supplementary material with the manuscript

Human research participants

Policy information about studies involving human research participants and Sex and Gender in Research.

Reporting on sex and gender	*Use the terms sex (biological attribute) and gender (shaped by social and cultural circumstances) carefully in order to avoid confusing both terms. Indicate if findings apply to only one sex or gender; describe whether sex and gender were considered in study design whether sex and/or gender was determined based on self-reporting or assigned and methods used. Provide in the source data disaggregated sex and gender data where this information has been collected, and consent has been obtained for sharing of individual-level data; provide overall numbers in this Reporting Summary. Please state if this information has not been collected. Report sex- and gender-based analyses where performed, justify reasons for lack of sex- and gender-based analysis.*
Population characteristics	*Describe the covariate-relevant population characteristics of the human research participants (e.g. age, genotypic information, past and current diagnosis and treatment categories). If you filled out the behavioural & social sciences study design questions and have nothing to add here, write "See above."*
Recruitment	*Describe how participants were recruited. Outline any potential self-selection bias or other biases that may be present and how these are likely to impact results.*
Ethics oversight	*Identify the organization(s) that approved the study protocol.*

Note that full information on the approval of the study protocol must also be provided in the manuscript.

Field-specific reporting

Please select the one below that is the best fit for your research. If you are not sure, read the appropriate sections before making your selection.

☐ Life sciences ☐ Behavioural & social sciences ☒ Ecological, evolutionary & environmental sciences

For a reference copy of the document with all sections, see nature.com/documents/nr-reporting-summary-flat.pdf

Ecological, evolutionary & environmental sciences study design

All studies must disclose on these points even when the disclosure is negative.

Study description	Analysis of archaeocyath species diversity, sizes, origination and extinction rates, reef sizes and their palaeodepth distribution, number of reefal palaeocommunities and reefal metacommunity from the lower Cambrian of the Siberian Platform.
Research sample	A sample set includes 5930 individuals representing 84 species, including 80 archaeocyaths and 4 associated coralomorphs and cribricyaths, from 53 lower Cambrian Stage 2 and 3 occurrences of the southern Siberian Platform (middle Aldan and Lena rivers). All the reef sites discovered in the area under study have been sampled. All the mature individuals being discovered in thin sections of these samples have being identified at the species level by AZ.
Sampling strategy	Bulk samples were collected from early Cambrian reefs of the Pestrotsvet and Perekhod formations, embracing approximately 11 million years interval. The principal sampling set was collected from three temporal transects (Tommotian 1 and Atdabanian 1 and 4) representing coeval reefs of different facies zones over a distance of 100 km. The sample size was determined by the equipment, which was used later on for the thin sectioning (about 10 to 10 cm). Because archaeocyths were small animals (about 1 cm in diameter in average) and about 10 samples of each reef site was collected, this sample size was enough to get a sampling set of a palaeocommunity.
Data collection	The original data collection was carried by A. Zhuravlev during field works of 1978-1987. Archaeocyath sponges in thin sections were identified at the species level and counted by AZ. All the data were subsequently plotted in Excel files.
Timing and spatial scale	Neither frequency nor periodicity of sampling are applicable to research with fossil material.
Data exclusions	No data were excluded.
Reproducibility	This was not an experimental study. All data and code are included in the SI so the results can be replicated.
Randomization	Randomization was not relevant to the study as it is not experimental in this way.
Blinding	Blinding was not relevant to the study as it is not experimental in this way.

Did the study involve field work? ☒ Yes ☐ No

Field work, collection and transport

Field conditions	River-banks on the Central Siberian Plateau, mostly larch taiga-type forest. These sites are fossil localities, so all relevant palaeoenvironmental information is included in the main text.
Location	Republic of Sakha (Yakutia), Russia. This site was not under water.
Access & import/export	N.a. No biological samples collected.
Disturbance	No disturbance for the nature was caused by the study because the rock sampling sites were naturally eroded by the rivers.

Reporting for specific materials, systems and methods

We require information from authors about some types of materials, experimental systems and methods used in many studies. Here, indicate whether each material, system or method listed is relevant to your study. If you are not sure if a list item applies to your research, read the appropriate section before selecting a response.

Materials & experimental systems

n/a	Involved in the study	
☒	☐	Antibodies
☒	☐	Eukaryotic cell lines
☐	☒	Palaeontology and archaeology
☒	☐	Animals and other organisms
☒	☐	Clinical data
☒	☐	Dual use research of concern

Methods

n/a	Involved in the study	
☒	☐	ChIP-seq
☒	☐	Flow cytometry
☒	☐	MRI-based neuroimaging

Palaeontology and Archaeology

Specimen provenance	The specimens were collected on the territory of the former Republic of Yakutia, USSR [=Republic of Sakha (Yakutia), Russian Federation] during 1978-1987. No special permissions for collecting were required during that period.
Specimen deposition	The specimens have been deposited at the Laboratory of Ancient Organisms, Borissiak Palaeontological Institute, Russian Academy of Sciences, Moscow, Russian Federation.
Dating methods	N/A

☐ Tick this box to confirm that the raw and calibrated dates are available in the paper or in Supplementary Information.

Ethics oversight	N/A

Note that full information on the approval of the study protocol must also be provided in the manuscript.
